# Editorial: Public health challenges in post-Soviet countries during and beyond COVID-19, volume II

**DOI:** 10.3389/fpubh.2026.1975308

**Published:** 2026-10-22

**Authors:** Antonio Sarria-Santamera, Ida Liseckiene, Natalya Glushkova, Yuliya Semenova

**Affiliations:** 1 Department of Biological and Biomedical Science, School of Medicine, Nazarbayev University, Astana, Kazakhstan; 2 Department of Family Medicine, Lithuanian University of Health Sciences, Kaunas, Lithuania; 3 Department of Public Health, Lithuanian University of Health Sciences, Kaunas, Lithuania; 4 Department of Epidemiology, Biostatistics and Evidence-Based Medicine, Al-Farabi Kazakh National University, Almaty, Kazakhstan; 5 Department of Surgery, School of Medicine, Nazarbayev University, Astana, Kazakhstan

**Keywords:** universal health coverage, health systems resilience, pandemic preparedness, health inequalities, Central Asia and Eastern Europe, health policy, COVID-19

On January 30, 2020, the WHO Director-General accepted the recommendation of the Emergency Committee and declared the novel coronavirus outbreak (2019-nCoV) a Public Health Emergency of International Concern (PHEIC) (1). Post-Soviet countries (PSC) faced significant challenges during the COVID-19 pandemic. As for many other countries, the pandemic was the first experience of such magnitude for Ministries of Health (MoH) since the fall of the Soviet Union in 1991. Therefore, many MoHs in the PSC lacked the necessary infrastructure, protocols, and staff to manage such events. However, while public health systems in many developing countries elsewhere are subpar, PSC inherited systems based on the Semashko model that were undergoing reforms to varying degrees (2). This factor proved critical to countries' capacity to respond to the pandemic, as public health emergency response systems had not been updated since Soviet times. These limitations are especially evident in the lack of progress in incorporating up-to-date methodologies of disease surveillance and laboratory testing. As a result, the COVID-19 pandemic became a system shock that challenged the readiness of public health emergency response capacities. While it is clear that PSCs were not ready (as in many developed countries), it is not clear whether the MoHs in these countries have learned their lessons and strengthened their capacities to face the next pandemic.

The COVID-19 pandemic had devastating impacts on public health, leading to the inaccessibility of health services for many people in need, catastrophic healthcare expenditures, and premature deaths. The countries experienced a shortage of facilities and medical professionals, a shortage that was particularly pronounced due to earlier reductions in hospital beds. Resources proved to be insufficient due to a severe shortage of essentials like medicines, diagnostic tests, and personal protective equipment, particularly during the peak period (3, 4).

Researchers from the region investigated aspects of public health systems such as a shift toward private healthcare. The example of Kazakhstan gives an interesting perspective for readiness as its healthcare system was based on the Soviet-era system, and the private healthcare sector was almost non-existent (Alibek et al.) (5). However, in the decade preceding the pandemic, healthcare shifted toward a higher number of private healthcare facilities. Thus, the pandemic required the government of Kazakhstan to mobilize both public and private healthcare providers to address the dramatic increase in patients. Although private hospitals were not originally adapted to manage large-scale infectious disease outbreaks, they adjusted by setting up COVID-19 units, at the cost of considerable business disruptions due to decreased patient flow and financial constraints.

The pandemic exacerbated many prior challenges in the healthcare system of PSC, including issues of trust and violence against healthcare workers: up to 38% of healthcare professionals experience some form of violence in the workplace (6). The COVID-19 pandemic brought not only increased workloads and burnout, but a threat of violence from patients and family members (7, 8). Moreover, the pandemic has eroded the societal respect traditionally given to healthcare workers (9): in Moldova, almost half of the violence aimed at healthcare workers primarily involved verbal threats; the COVID-19 pandemic increased concerns about workplace violence (Ciobanu et al.).

COVID-19 infection brought new attention to chronic diseases, including cardiovascular disease and type 2 diabetes mellitus. While these diseases traditionally affect older populations, their prevalence among younger populations is increasing (10). Moiynbayeva et al. forecast a rise in cardiovascular disease and stroke prevalence in Kazakhstan, Kyrgyzstan, Uzbekistan, Tajikistan, and Turkmenistan. This is concerning, as patients with chronic diseases were at a higher risk of severe complications and death from COVID-19 (11–13).

The pandemic's demographic effects were significant worldwide, though different countries reacted differently to lockdowns, declining birth rates, reduced in-person communication, fear of COVID-19, and other issues. Karibayeva et al. report no long-term effect on birthrate in Kazakhstan, which might suggest a limited effect of the pandemic on life. However, long-term studies on birthrate and reproductive health are needed, as some studies suggest that COVID-19 infection might have long-term effects on the reproductive system, including hormonal disturbances that can affect regularity of the menstrual cycle, ovulatory function, and receptivity of the endometrium (14).

Data from United States suggests that the COVID-19 pandemic has worsened existing health disparities and disproportionately affected racial and ethnic minorities (15). As reported by the Centers for Disease Control and Prevention, segregated ethnic groups have experienced higher rates of infection, hospitalization, and death compared to other populations (16). These findings were corroborated by European studies (17, 18), which report that people living in segregated, low-income neighborhoods were affected by disproportionately higher rates of COVID-19 infections. One of the other vulnerable groups are older adults, particularly 65 and over, who were at the higher risk for severe complications and death from COVID-19 and thus requiring improved access to healthcare services for both acute and chronic conditions (19). A survey of 1,503 Lithuanians aged 65 and over (Kalibatas et al.) reports that the primary reasons for non-utilization of healthcare services included fear of COVID-19 infection for 1 in 6 respondents. These studies provide further support for the view that the COVID-19 pandemic had a significant effect on healthcare systems worldwide, limiting access to routine healthcare and exacerbating inequalities in access to healthcare (20).

Health-related behaviors in older adults were also affected during and after the COVID-19 pandemic: in Lithuania, there was a decrease in physical activity, face-to-face communications, and an increase in remote communication in older adults in Lithuania. Factors like education, income, age, residence, and health status affected these outcomes, suggesting that a differential approach is needed for mitigating negative outcomes from the pandemic (Sauliune et al.).

While the WHO declared the end of the COVID-19 pandemic in 2023, populations across the world were reminded of dangers posed by fast spreading and deadly viruses. Public attention to emerging viruses was at all time high during and several years after the pandemic, but the challenges associated with managing a large epidemic are still potentially there. While thousands of research papers were dedicated to the COVID-19 pandemic, much lesser attention is given to lessons learned from the pandemic, or state of readiness in case of another worldwide outbreak of infectious disease.

One of the significant challenges in PSC during the pandemic was undiagnosed infection. The potential undercount of overall cases has been estimated to be by 55%−60% by official statistics (21), although managing the COVID-19 pandemic may have also decreased healthcare systems' ability to surveil and report other infectious diseases (24).

COVID-19 pandemic also brought new attention to hygiene among different population groups, including children (22, 23): the majority of school children in Karaganda region, Kazakhstan, showed appropriate hand hygiene practices, though only one-fourth of the students washed hands after contact with a sick friend. Therefore, to reduce the spread of infectious disease among school children, public health interventions should focus on behavioral reinforcement in addition to education, including habit formation techniques and peer modeling (Bolatova et al.).

Overall, studies from the impact of the COVID-19 pandemic exposed gaps in the PSC public health capacity and pandemic preparedness, reflect on risks associated with health delivery services, violence against health workers, and population behavior, as well as the impact of COVID-19 on the intersection of chronic and infectious diseases.

However, the studies were predominantly cross-sectional designs and data on long-term public health impacts or the persistent effects of the COVID-19 pandemic or comparison of health systems pre- and post-pandemic period are lacking. Globally, lessons from the pandemic have emphasized the need for comprehensive health policies that strengthen national health systems on the foundations of public health and universal health coverage, focusing on the management and prevention of both chronic and infectious diseases while maintaining a strong emphasis on population health behavior, especially among vulnerable populations. Countries should determine and expand national pandemic preparedness plans to prevent and respond to newly emerging infectious diseases.
